# Lighting the way: how the *Vibrio fischeri* model microbe reveals the complexity of Earth’s “simplest” life forms

**DOI:** 10.1128/jb.00035-24

**Published:** 2024-05-02

**Authors:** Alecia N. Septer, Karen L. Visick

**Affiliations:** 1 Department of Earth, Marine and Environmental Sciences, University of North Carolina, Chapel Hill, North Carolina, USA; 2 Department of Microbiology and Immunology, Loyola University Chicago, Maywood, Illinois, USA; Geisel School of Medicine at Dartmouth, Hanover, New Hampshire, USA

**Keywords:** *Vibrio fischeri*, biofilms, motility, type VI secretion, symbiosis, luminescence

## Abstract

*Vibrio* (*Aliivibrio*) *fischeri*’s initial rise to fame derived from its alluring production of blue-green light. Subsequent studies to probe the mechanisms underlying this bioluminescence helped the field discover the phenomenon now known as quorum sensing. Orthologs of quorum-sensing regulators (i.e., LuxR and LuxI) originally identified in *V. fischeri* were subsequently uncovered in a plethora of bacterial species, and analogous pathways were found in yet others. Over the past three decades, the study of this microbe has greatly expanded to probe the unique role of *V. fischeri* as the exclusive symbiont of the light organ of the Hawaiian bobtail squid, *Euprymna scolopes*. Buoyed by this optically amenable host and by persistent and insightful researchers who have applied novel and cross-disciplinary approaches, *V. fischeri* has developed into a robust model for microbe-host associations. It has contributed to our understanding of how bacteria experience and respond to specific, often fluxing environmental conditions and the mechanisms by which bacteria impact the development of their host. It has also deepened our understanding of numerous microbial processes such as motility and chemotaxis, biofilm formation and dispersal, and bacterial competition, and of the relevance of specific bacterial genes in the context of colonizing an animal host. Parallels in these processes between this symbiont and bacteria studied as pathogens are readily apparent, demonstrating functional conservation across diverse associations and permitting a reinterpretation of “pathogenesis.” Collectively, these advances built a foundation for microbiome studies and have positioned *V. fischeri* to continue to expand the frontiers of our understanding of the microbial world inside animals.

## INTRODUCTION

Life is thought to have evolved in the ocean before expanding to terrestrial habitats. It is no wonder then that marine bacteria share genes, physiology, functional pathways, and regulatory mechanisms with their terrestrial cousins. These pathways and processes represent deeply rooted, conserved strategies that have evolved over billions of years and adapted to new habitats as the Earth and its increasingly diverse host species changed over time.

The story of *Vibrio* (*Aliivibrio*) *fischeri* starts in the ocean. Although *V. fischeri* is a rare member of the seawater community (<0.1%) (1–3), it can be easily cultured. It was on these growth plates that researchers like Beijerinck and Fischer first noticed this bacterium’s intriguing ability to glow [reviewed in reference (4)]. It is indeed a wonder—biologically produced light! One that we are first captivated by as children when we see fireflies or bioluminescent waves in the ocean. However, *V. fischeri* had more secrets to reveal than the source of its internal illumination. Indeed, it was in *V. fischeri* where “quorum sensing”—the detection of the presence of “like” bacteria—was first discovered, which is now understood to be a widespread bacterial phenomenon (5). This organism was later used to identify or study many other conserved mechanisms, such as those for interbacterial cooperation, competition, and host colonization.

Although *V. fischeri* can be cultured from symbiotic organs found in diverse fish and squid species across the globe (6), it is best known for its exclusive ability to colonize the light organ of the Hawaiian bobtail squid *Euprymna scolopes* (7, 8). As a beneficial symbiont, *V. fischeri* bucks the dogma associated with the study of pathogens by producing many of the same types of molecules and using behaviors often labeled as “pathogenic.” For example, despite that there is no known ability of *V. fischeri* to cause disease in any host, it produces bacterial surface molecules, previously designated as pathogen-associated molecular patterns, which stimulate beneficial morphological changes in the symbiotic host, prompting the field to rename them as MAMPs (microbe-associated molecular patterns) (9). Moreover, while *V. fischeri* causes no harm, it produces the same toxin molecule, tracheal cytotoxin (TCT), which disrupts respiratory mucosa when produced by the human pathogen, *Bordetella pertussis* (9–12). *V. fischeri* also engages in other well-studied behaviors often associated with pathogenesis, including motility, biofilm formation, and type VI secretion. Using *V. fischeri* to examine these “pathogenic” traits in the context of a beneficial symbiosis has permitted the field of microbiology to better understand how microbes in general interact with the eukaryotic world and has contributed to the foundation needed for the development of microbiome studies.

This review starts with the initial isolation of *V. fischeri* in the late 1800s and continues through a subset of discoveries made with this model organism over the past 130 years, including a few serendipitous events. Through reflections on *V. fischeri* history and discoveries, we hope to highlight the important scientific breakthroughs made in *V. fischeri* and its potential to contribute to new revelations in host-microbe interactions, environmental signal transduction, and ecological and evolutionary dynamics.

## EARLY HISTORY

**Fig 1:**
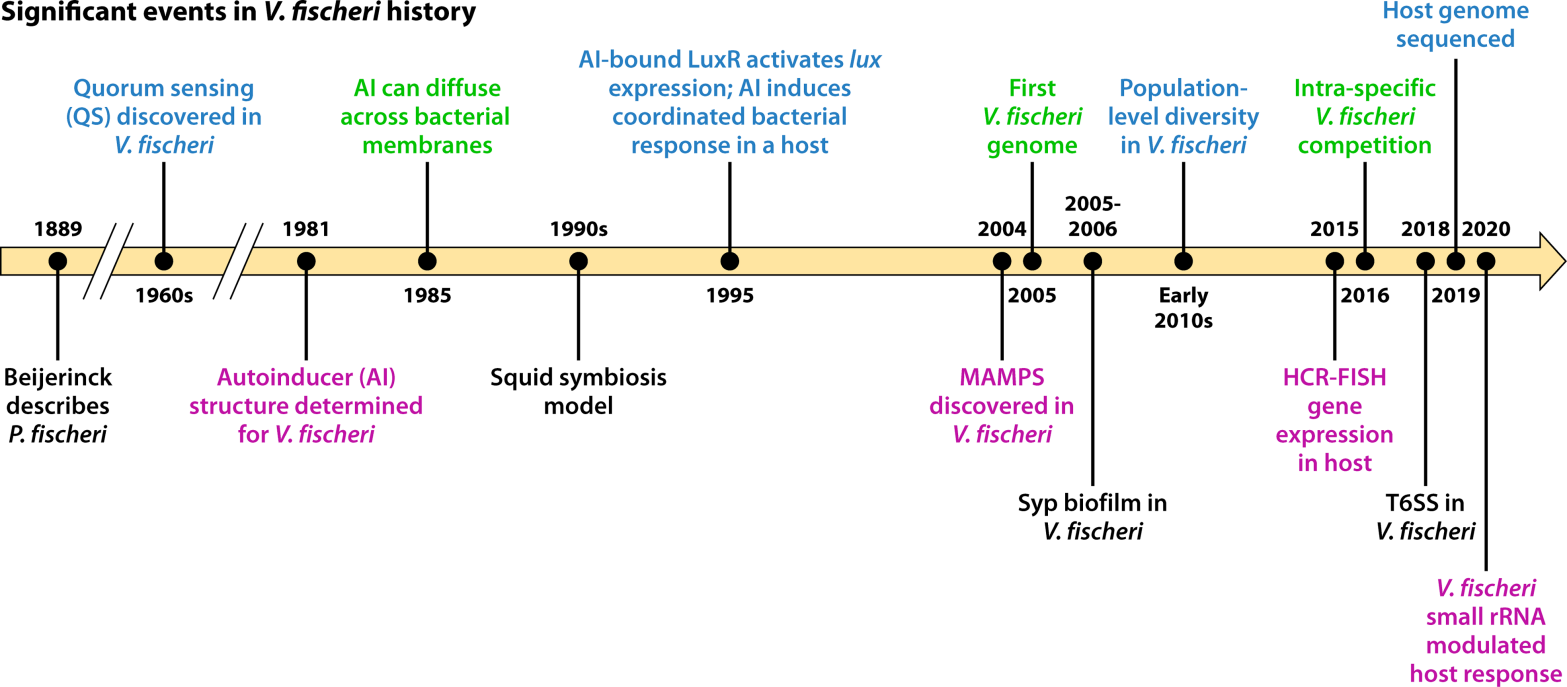
Time line of *V. fischeri* history. Key events and discoveries made using *V. fischeri* as a model microbe.

*V. fischeri* isolates were first described as early as the late 1800s by researchers such as Martinus Beijerinck and Bernhard Fischer, who studied bioluminescent bacteria (Fig. 1). Originally described in 1889 by Beijerinck as *Photobacterium fischeri* (13), the name and taxonomy changed many times over the following 130 years as new strains were isolated, characterized, and compared to the growing number of related Vibrionaceae. The early history of the discovery of *V. fischeri* is nicely described in a review of Beijerinck by Robertson et al. (14). Although *V. fischeri* was again recently moved into a new genus (*Aliivibrio*) (15), many researchers choose to retain the *Vibrio* designation or name both genera in publications to underscore the evolutionary relationship between *V. fischeri* and other well-characterized *Vibrio* species, while still acknowledging the distinction between *V. fischeri* and other Vibrionaceae.

**Fig 2:**
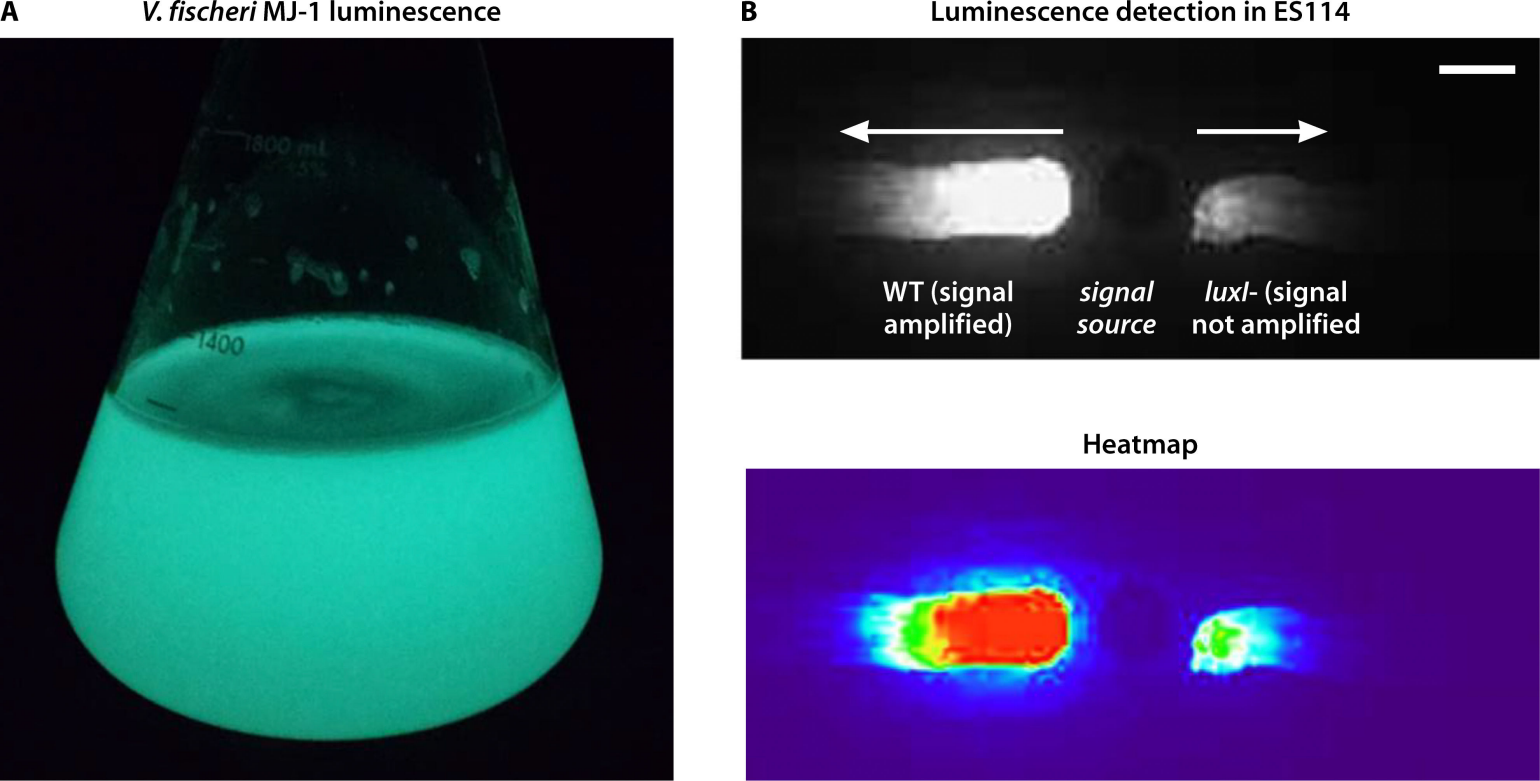
*V. fischeri* luminescence. (**A**) Flask of glowing MJ-1 culture. (**B**) Autoinducer 3-oxo-C6 produced by *arcA* mutant donor (signal source) diffuses to induce luminescence in adjacent wild-type and *luxI* mutant cells. The top image shows luminescence, and the bottom image is a heatmap of luminescence intensity using the Thermal setting in FIJI.

The 1960s and the ensuing two decades saw a resurgence of interest in bioluminescent marine bacteria by researchers such as J. Woodland Hastings, Edward Meighen, Kenneth Nealson, E. Peter Greenberg, Michael Silverman, Bonnie Bassler, and others. Isolates of *V. fischeri* and *Vibrio harveyi* were heavily investigated for biochemistry and, later, genetics underlying luminescence properties. These studies documented the behavior of light production in culture, showing that it occurred after a lag relative to the growth of the bacteria. We now understand that this phenomenon is mediated by the release and subsequent uptake of and response to a signaling molecule, autoinducer (16, 17). This mechanism, known as quorum sensing, permits the bacteria to sense when there are sufficient numbers of similar bacteria to turn on the expression of group-behavior genes such as those for bioluminescence (5). The study of light production in *V. fischeri* was carried out in a variety of *V. fischeri* strains, including MJ-1, a brightly luminous strain from the bioluminescent organ of the fish *Monocentris japonica* that produced large amounts of autoinducer (17, 18). Its readily visible display of blue-green light made MJ-1 an appealing model microbe to study (Fig. 2A).

## LUMINESCENCE AND QUORUM SENSING

The early history of luminescence and quorum sensing has been nicely summarized in a Journal of Bacteriology guest commentary (19), and underlying mechanisms are described in more detail in a Microbiological Reviews article (20). Key early discoveries about luminescence and quorum sensing were generated through the use of both the brightly luminous MJ-1 and strain B61, an isolate that made little autoinducer but could respond to it, providing a valuable bioassay; using these strains together permitted researchers to monitor the production of autoinducer in strain MJ-1 and its role in inducing bioluminescence in strain B61 (21). The structure of the first autoinducer, the small, secreted molecule that was known to activate light production, was first identified from *V. fischeri* as an acyl-homoserine lactone (AHL), specifically *N*-(3-oxohexanoyl)-L-homoserine lactone (HSL) or 3-oxo-C6-HSL (17). It was subsequently shown that autoinducer can diffuse across bacterial membranes in culture, explaining how its accumulation could drive changes in gene expression (22).

While *V. harveyi* was and is also used as a model to identify and understand the genetic determinants of quorum sensing, the genomic arrangement of regulatory factors in *V. fischeri* allowed for work in this organism to initially progress faster. In *V. fischeri*, the locus that controlled its light production, *lux*, also included *lux* regulators, which turned out not to be the case for *V. harveyi* (23, 24). As a result, the *V. fischeri lux* locus could be cloned as a unit into *Escherichia coli*, which permitted that microbe to produce bioluminescence in a cell-density-dependent manner. Indeed, this work helped uncover gene function, for example, identifying LuxI as the autoinducer synthase (23, 25). The LuxI-produced 3-oxo-C6 was later shown to bind the N-terminal domain of the transcription factor LuxR to activate the expression of the *lux* locus (26).

The direct regulatory connection between quorum-sensing mechanisms and light production, which serves as a natural reporter for quorum sensing, established *V. fischeri* as a key model system for further studies of this phenotype. This work also laid the foundation for the discoveries [reviewed in reference (5)] that (i) bacteria produce a diversity of AHLs by LuxI-like proteins that can be detected by and bound to LuxR-like proteins, (ii) the *luxI/luxR* homologs control a variety of other traits, including virulence factors and plasmid transfer, in response to cell density (or, more specifically, levels of autoinducer), and (iii) bacteria can recognize autoinducer molecules that are self-made (same species) as well as those that are not (different species) to drive appropriate responses (5).

New insights into quorum-sensing mechanisms and regulation emerged as *V. fischeri* strain ES114, an isolate from the light organ of *E. scolopes*, was studied both in culture and within its natural host. Because *V. fischeri* was well-known at this point as a bioluminescent microorganism, it was of some surprise to researchers when isolates from squid, including ES114, formed colonies that appeared non-luminous, or dark, on petri plates (27). It turned out that ES114 and other squid-derived isolates are competent to produce light in culture and on petri plates but are considered “dim” because they produce light below the level that can be observed with the naked eye. Light production is induced over 1,000-fold during symbiosis relative to *in vitro* measurements, a phenomenon that reinforced the idea that the light organ environment differs in important ways from laboratory culture (27) and could be leveraged to understand how bacteria sense changes in their surroundings to regulate functions during habitat transition. In fact, measurements of autoinducer production in the squid symbiosis provided the first direct evidence that bacterial colonization of animal tissue does indeed lead to autoinducer production and accumulation sufficient to induce a coordinated bacterial response such as light production (28). In addition, host colonization experiments revealed that autoinducer can diffuse between colonization sites (i.e., crypts) to induce luminescence in cells physically separated by host tissue (29).

Environmental conditions also regulate *lux* gene expression in *V. fischeri*, underscoring the importance of considering both the physiochemical properties of the cells’ environment and their metabolic state. For example, it was determined that the redox-controlled regulator ArcA negatively controls light production in culture; when the *arcA* gene is disrupted, light production by ES114 rises about 500-fold, to the level of detection by the naked eye (30). Deletion of *arcA* results in derepression of *luxI*, promoting light production at lower cell density. Furthermore, the high levels of autoinducer produced by this strain are sufficient to induce luminescence in neighboring ArcA-repressed cells (Fig. 2B) (29). These findings suggest that autoinducer molecules may also communicate information about environmental conditions (e.g., redox), in addition to cell density, prompting some to prefer the term “pheromone signaling” over “quorum sensing” to be more inclusive in describing the role of this signaling molecule in regulating cellular behavior and function (31). Regardless of terminology, it is clear that *V. fischeri* has evolved to use this signaling mechanism to do more than simply sense a quorum.

Finally, although luminescence regulation in *V. fischeri* initially appeared more straightforward than its *V. harveyi* relative, later work revealed that this was not the case. *V. harveyi* produces and responds to three autoinducers via specific sensor proteins that together control the activation of a downstream response regulator protein, LuxO. Activated LuxO promotes the transcription of genes for small regulatory RNAs (sRNAs) that prevent the synthesis of a *lux*-specific transcription factor, LuxR, which in turn induces the transcription of the *lux* operon [reviewed in reference (32)]. That this downstream transcription factor was, unfortunately, termed LuxR has been a source of confusion in the literature as the *V. harveyi* protein is not homologous to the *V. fischeri* protein. To avoid additional confusion, yet adding to it, homologs of the *V. harveyi* LuxR regulator have been given a variety of other names in the literature, including LitR (*V. fischeri*), HapR (*Vibrio cholerae*), and SmcR (*Vibrio vulnificus*), to name a few (33). In *V. fischeri*, LitR production is managed via an upstream regulatory scheme similar to that of *V. harveyi*, with two autoinducers (distinct from that produced by LuxI) that indirectly control the activity of the LuxO protein that, in turn, induces the transcription of a LitR-inhibitory sRNA. LitR interfaces with the LuxRI regulatory pathway by controlling *luxR* transcription, thus providing another level of control over light production (34–37). In addition, new *lux* regulators have been identified recently (38). It is clear that *V. fischeri* is not simple and that the book is not yet closed on understanding how *V. fischeri* controls its light production either in the lab or in the context of symbiosis.

## *V. FISCHERI* IN SYMBIOSIS

**Fig 3:**
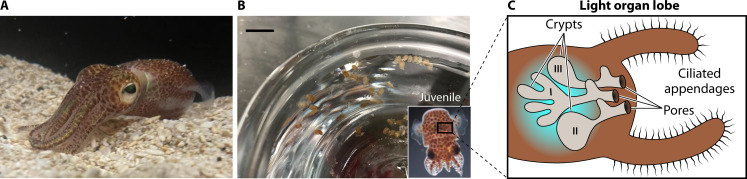
*V. fischeri*’s symbiotic host, the squid *E. scolopes*. (**A**) Adult *E. scolopes* (photo by Vanya Tepavčević, reproduced with permission). (**B**) Juvenile *E. scolopes* in bowl (photo by Andrea Suria); inset, single juvenile (photo by Val Ray, reproduced with permission). (**C**) Drawing of the light organ of a juvenile squid.

In the late 1980s, when work on the *V. fischeri-E. scolopes* symbiosis began, there were few model systems to study the interactions between a beneficial bacterium and an animal host, whether marine or terrestrial. Although researchers had a long history of using marine animals as model systems to study neurobiology, embryology, and aging (39, 40), the role of bacteria in shaping the development and survivability of marine hosts was underexplored. Thus, early experiments that colonized juvenile squid with *V. fischeri* revealed great potential for this marine model system to fill an important knowledge gap in symbiosis research (Fig. 3). These pioneering efforts were carried out by Margaret McFall-Ngai and Ruby (see Box 1). These researchers were influenced by a seminal study by Wei and Young (41) on the initiation of this symbiosis and were aided by studies that provided insight into the light organ anatomy of related squid species (42, 43). This elegant work resulted in a natural host system for an already well-known and valuable bacterial model species, *V. fischeri*. The continued insight and dedication of McFall-Ngai and Ruby and their scientific progeny shaped this system into an invaluable tool for understanding the microbial world within animals.

**Fig 4:**
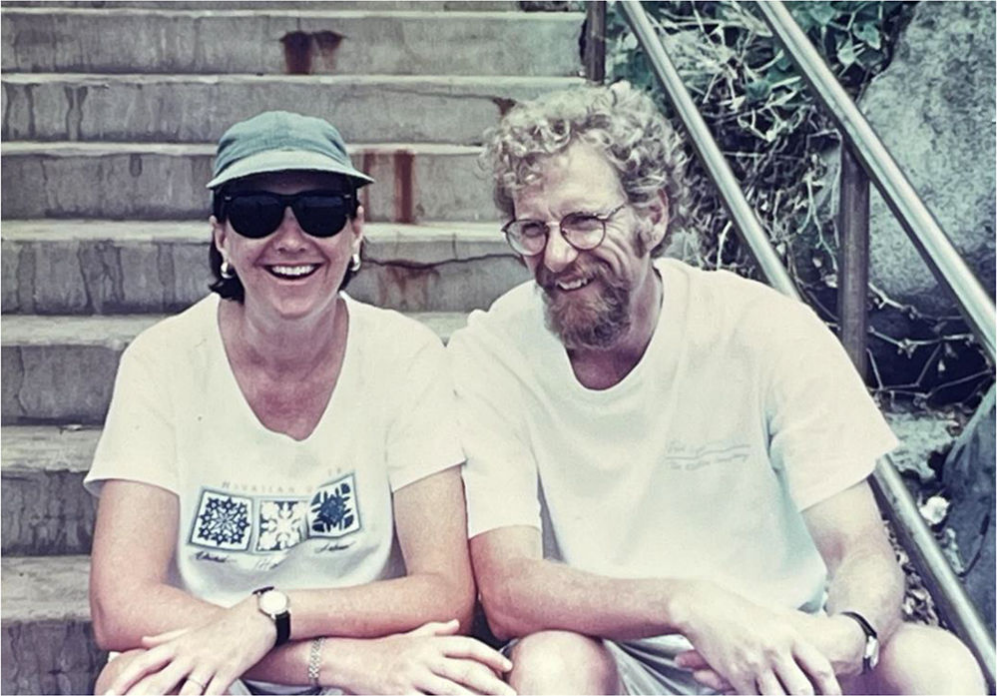
Margaret McFall-Ngai and Ned Ruby. Circa 1996–1997; sitting on the steps leading into the ocean beside the Kewalo Marine Laboratory, University of Hawaii-Manoa, Honolulu, Hawaii.

Box 1 Today, *V. fischeri* is best studied as the exclusive symbiont within the light organ of the Hawaiian bobtail squid *E. scolopes* due to the pioneering research of Drs. Margaret McFall-Ngai and Edward (Ned) Ruby. Early in her Ph.D. studies with Dr. James Moran at the University of California, Los Angeles (UCLA), McFall-Ngai developed interests in two areas: (i) tissues that interact with light, such as the eye and notably the light-emitting organs (light organs) of fish and squid, and (ii) symbioses of animals with bacteria. Those interests continued during her two post-doctoral positions, working with Dr. Joseph Horwitz at the Jules Stein Eye Institute at UCLA and, later, with Dr. George Somero at Scripps Institution of Oceanography, University of California, San Diego. During that time, McFall-Ngai began to look for a model system that would permit her to integrate those interests. Her selection of *E. scolopes*, the Hawaiian bobtail squid, came about from a combination of serendipitous events and practicality. It was necessary to find a light-organ-containing host species that could be accessible in the field, easily maintained in the lab, bred, and experimentally colonized with its symbiont. Many of the light-organ-containing *Euprymna* and *Sepiola* squid species are found in hard-to-access habitats, such as deep, cold waters, and fish species have only rarely been bred in captivity and/or experimentally colonized (44). However, a colleague of McFall-Ngai’s suggested she explore the Hawaiian bobtail squid, which could be caught easily at night in knee-deep water using a flashlight and dipnet. As McFall-Ngai investigated the potential of this system, she realized that its study would greatly benefit from engendering another symbiosis, a partnership with a scientist who could balance her animal perspective and provide expertise on the bacterial side of this association. Ultimately, she recruited Ruby to the work (Fig. 4). Ruby brought to the system substantial strengths in bacterial physiology as well as experience in *Vibrio* biology and bacterial bioluminescence, as he had studied *V. fischeri* (*Photobacterium fischeri*) and other luminous bacteria in both his graduate work with Dr. Kenneth Nealson at Scripps Institution of Oceanography and post-graduate work with Dr. J. Woodland (Woody) Hastings at Harvard University. Together, McFall-Ngai and Ruby discovered that the Hawaiian bobtail squid could thrive and be bred in lab aquaria, where females lay clutches of eggs from which hundreds of animals would hatch (Fig. 3B)—a clear advantage of this system over vertebrate models. The animals hatched without their symbiont, which could be added back to the water to colonize juveniles through natural infection routes. All field-caught animals contained their *V. fischeri* symbiont and, correspondingly, colonized experimental squid maintained their symbionts over their lifespan. Like the *V. fischeri*-squid symbiosis, the human partnership has been a long-term and productive relationship with over 35 years of scientific cooperation.

Beyond their initiative in establishing this model system, McFall-Ngai and Ruby can be truly considered innovators in both thought and approach. Over the years, they have sought out and applied numerous new technologies and/or scientific advances to the study of the system, including but not limited to genomic sequencing of both organisms, application of microarray technology, and then later other transcriptomic approaches to understand symbiosis-specific gene regulation, use of fluorescent proteins and imaging to visually observe the position and dynamics of the symbiont during host colonization, and hybrid chain reaction fluorescence *in situ* hybridization (HCR-FISH) to evaluate host and symbiont gene expression *in situ*, to name a few. McFall-Ngai and Ruby are also highly collaborative, recruiting scientists from diverse fields to approach important questions from new and different perspectives. Their infectious enthusiasm, open sharing of unpublished data, and generosity in giving credit are just some of their engaging qualities that draw others into the work. Furthermore, they have been remarkable cheerleaders for the system, a fact that has promoted the interest and support of multiple funding sources. Finally, as a testament to their love of science and the model system that they established, McFall-Ngai and Ruby hold an annual conference for their labs and the labs of their scientific progeny to permit an integrated understanding of the symbiosis and to facilitate communication between members of the field. While the annual conference may now be organized by members of the greater community, there is no doubt of the impact that McFall-Ngai and Ruby continue to make as they proceed to ask deep and thought-provoking questions and contribute keen insight and perspective that guide the field to the next levels of engagement and discovery.

Early ecological studies of Kāne‘ohe Bay, Hawaii, and the surrounding ocean revealed important insights about symbiont abundance and transmission. Experiments in the 1990s to estimate symbiont levels in seawater showed significantly higher *V. fischeri* concentrations in areas where squid were found, relative to offshore water outside the bay (45). Indeed, exposing juveniles in the lab to seawater from Kāne‘ohe Bay vs offshore water revealed that only Kāne‘ohe Bay had sufficient levels of symbiont to permit the horizontal transmission of the symbiosis to the next generation. The reason, it would seem, has to do with adult behavior. Although squids are not known to remain close to their egg clutches like some octopus species, *E. scolopes* adults vent 90%–95% of their light organ content each dawn, which is thought to seed the environment for the next generation of hosts (45, 46). This finding revealed a connection between host behavior and symbiont transmission, a similar mechanism that has been observed in the establishment of human microbiomes and disease transmission (47, 48).

One key advantage of the squid-vibrio model system is the ability to non-invasively monitor colonization dynamics over time via light production, as high levels occur only when the bacteria have successfully grown inside the light organ. The presence of bacteria can be confirmed, and the specific number quantified, via homogenization and plate counting at the end of the experiment. Another key advantage is that the animal’s translucent tissue makes the squid amenable to direct visualization of the *V. fischeri* within. Furthermore, over the years, insights into colonization mechanisms have been facilitated by technological advances and tool development. For example, approaches such as the use of fluorescent proteins and optical sectioning with confocal microscopy have facilitated assessments of bacterial localization, gene expression, and the impact of *V. fischeri* on host development. This ability to directly observe the biogeography of *V. fischeri* and its impact on host cells within microhabitats provides a much more detailed picture of symbiosis than simple CFU measurements could.

Interdisciplinary collaborations have also greatly enhanced the utility of *V. fischeri* as a model organism, and we highlight a few here. Collaboration with engineers revealed the biophysics underlying how *V. fischeri* cells are entrained in fluid flow fields generated by the juvenile host to promote colonization (49). Mathematical modeling has been applied to both autoinducer diffusion dynamics (50) and type VI secretion system (T6SS) mediated interbacterial competition (51), allowing researchers to further explore the impacts of altering parameters *in silico* in a way that is not possible with biological experiments. Chemists have applied their imaging mass spectrometry approach (52) to reveal details about the chemical landscape of bacterial colonies and in symbiosis. Protein biochemists have explored symbiotically relevant signal transduction systems (53), and omics experts have applied their skills to explore the proteomes and transcriptomes of *V. fischeri* [e.g., references (54–56)].

The culmination of decades of work on *V. fischeri* and interdisciplinary collaborations results in a working model for how this organism colonizes and impacts host development. Two recent reviews describe this work in more detail (7, 8). Incredibly, despite the diversity and abundance of marine bacteria present in the ocean, *V. fischeri* is the sole microbe that can be cultured from the light organ of *E. scolopes*. The explanation for this extreme specificity appears to depend on multiple factors, including the ability of *V. fischeri* to resist innate immune host defenses such as phagocytic cells and molecules like nitric oxide (46, 57, 58). Newly hatched juvenile squid contain an immature version of the light organ that lacks *V. fischeri*, requiring them to recruit their symbiont from the surrounding seawater (Fig. 3). Rapid ventilation by the squid sweeps bacteria-containing seawater through the mantle cavity and across the light organ. Cilia on the surface of external appendages direct the fluid flow, channeling bacteria to quiet zones (49) where they accumulate in a biofilm-like aggregate (59). *V. fischeri* cells then use flagella- and chemotaxis-driven migration to enter pores, traverse ducts and antechambers, and navigate through bottlenecks to reach deep crypt spaces where they colonize (60–62). Three pores exist on each side of the bi-lobed organ, ultimately leading to three sets of crypts with different properties. The largest, crypt 1, is the most developed upon hatching, while crypt 3 is the smallest and least developed. At a minimum, these features permit colonization by multiple strains. Moreover, they indicate different environmental conditions that may impact bacterial physiology. Indeed, *V. fischeri* present in different crypts—and even in different regions within crypts—can have different levels of *lux* gene expression (63). Furthermore, crypts appear to have distinct amounts of nutrients such as cystine (64), and heterologous concentrations of phosphate exist within different crypt microenvironments (65). These studies of biogeography continue to provide important insights into the specific signals and environments experienced by *V. fischeri* and its corresponding responses.

*V. fischeri* rapidly exerts a substantial impact on the development of its host’s light organ. Within a short 3 hours of exposure, a small number of *V. fischeri* cells have attached and induced changes in host gene expression and trafficking of hemocytes, the squid’s phagocytic immune cells (66, 67). By 12 hours of colonization, bacterial MAMPs, including lipopolysaccharide (LPS) and TCT, derived from peptidoglycan, trigger other developmental events, including apoptosis of cells within the ciliated surface appendages and the subsequent regression and loss of those appendages (9, 68). These findings suggested that the primary function of the surface appendages is to facilitate bacterial colonization. Other developmental events induced by the bacteria occur, including duct narrowing caused by the polymerization of actin within host epithelial cells (69). Most recently, it was documented that the quorum sensing regulators LuxI and LuxR, but not light itself, are required to promote the constriction of bottlenecks between the antechambers and the deep crypt spaces (62, 70, 71). These bacteria-induced developmental changes highlight the intricate relationship between these two organisms and underscore the dynamic nature of diverse microenvironments that *V. fischeri* experiences.

Finally, although the bioluminescence likely provides camouflage to its host by means of counter-illumination during its nocturnal behavior (72), it remains uncertain exactly what advantage the light reaction provides to the bacteria. Mutants that cannot produce light are gradually lost from the symbiosis (73, 74), suggesting that the light reaction enhances the survival of symbiotic cells in the light organ. Furthermore, in mixed strain experiments, a “dark” (Δ*lux*) mutant is ultimately displaced by the wild-type parent, indicating that the loss of luminescence capability is detrimental regardless of the presence of a light-producing strain; the mutant cannot “freeload” or be protected by the presence of the wild-type strain (74). Several possibilities to account for the requirement of light production during symbiosis have been proposed that are not necessarily mutually exclusive: (i) the host may sense the lack of light and reject the symbiont through some mechanism, (ii) the light reaction, which consumes oxygen, may reduce oxygen levels to provide some protection against the generation of reactive oxygen species, and (iii) blue light may impact host cell function similar to the photomodulation effect it has on re-epithelialization of wounds in humans (75). Collectively, these findings show that *V. fischeri* endures biochemical and biophysical challenges in its host, similar to other beneficial and pathogenic bacteria; therefore, *in vivo* studies of *V. fischeri* behavior, physiology, and regulation have applications across diverse host-microbe associations.

## DEVELOPMENT OF GENETICS

**Fig 5:**
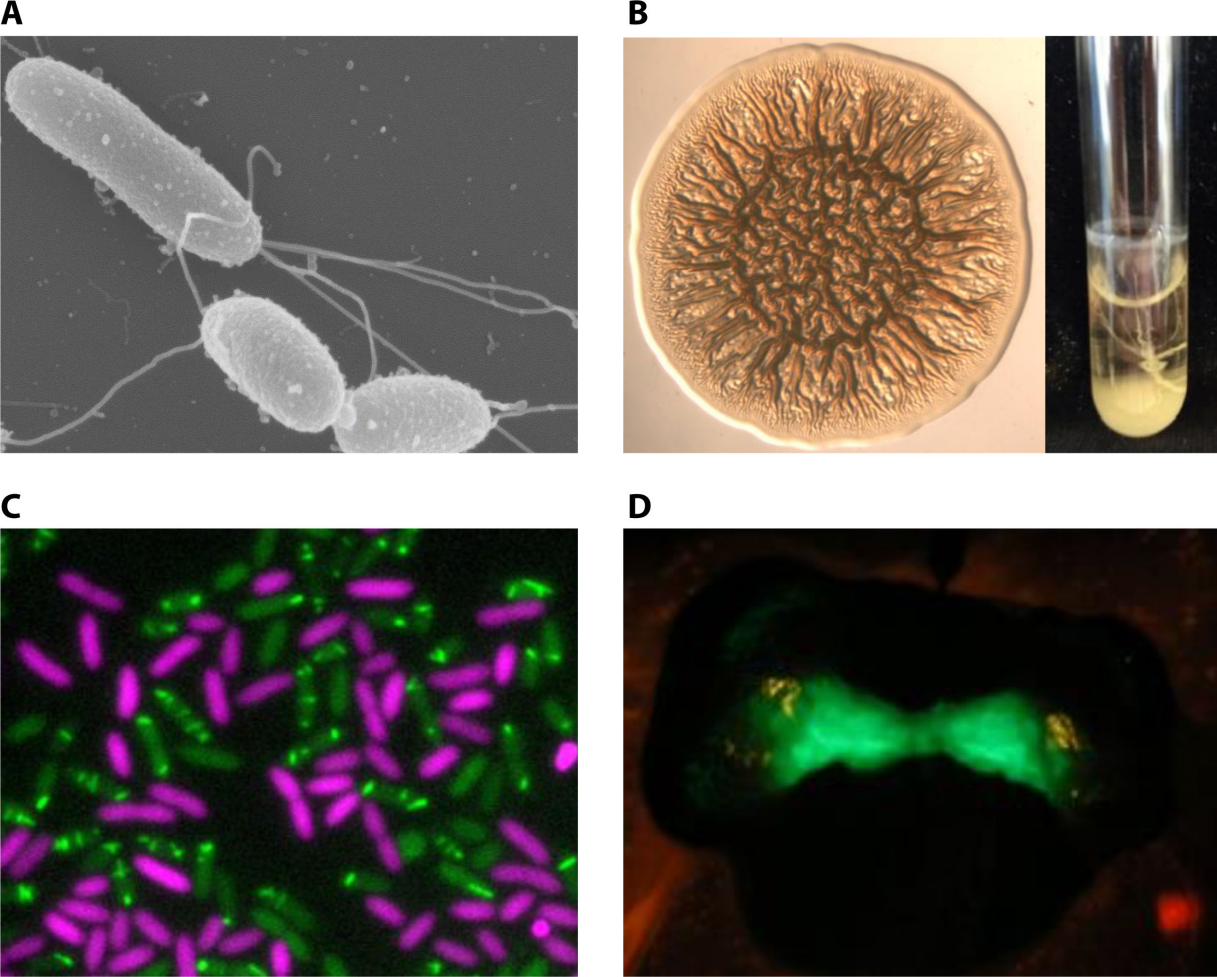
Select *V. fischeri* phenotypes. (**A**) Motility. *V. fischeri* cell with a tuft of polar flagella (Image courtesy of Stephanie Smith, reproduced with permission.). (**B**) Biofilm. (Left) Wrinkled colony formed by *rscS*-overexpressing, biofilm-induced *V. fischeri* strain. (Right) Biofilms formed by a biofilm-induced *V. fischeri* strain under shaking culture conditions. (**C**) T6SS-mediated competition. T6SS-expressing *V. fischeri* (green structures) in contact with target bacteria (magenta) (Image courtesy of Stephanie Smith, reproduced with permission.). (**D**) *E. scolopes* juvenile colonized with a GFP-based promoter reporter. Image captured using a dual filter to visualize the GFP from symbiotic cells and the red autofluorescent squid tissue.

For any model microorganism, the potential for discovery is greatly enhanced if it is genetically tractable. In the case of *V. fischeri*, although it took many years to develop the methodology that turned strain ES114 from “OK” to robustly tractable, the ability to connect genotype to phenotype further expanded its utility as a model organism. As with most bacteria, success was achieved after a series of trial and error. For example, early work identified a transducing phage that was used and then lost (76, 77). Conjugation was and continues to be a reliable method for the introduction of plasmids, both those that can replicate and those that cannot, the latter of which facilitated gene replacement approaches and transposon mutagenesis, both random (Tn*5*, Tn*10*, and Mariner) and site-specific (Tn*7*) (61, 78–85). The introduction of plasmids that carried *gfp* (green fluorescent protein) and other fluorescent markers opened new applications including visualizing the dynamics of *V. fischeri* colonization and gene expression [e.g., reference (59)] (Fig. 5). Highly stable plasmids (the pVSV series) were developed following the sequencing and characterization of a native *V. fischeri* plasmid, pES213, by combining the native origin of replication with other useful features such as an antibiotic resistance cassette, a multiple cloning site, and a fluorescence marker (63, 86). These pVSV plasmids have proven useful in numerous other bacterial species as well [e.g., reference (87, 88)]. Furthermore, these studies demonstrated that conjugal transfer of pES213 and, likely other plasmids, naturally occurs between strains in the context of symbiosis (86).

While conjugation remains a reliable approach to introducing plasmid DNA into *V. fischeri*, transformation has become preferred for other genetic manipulations (89). Work with *V. cholerae* determined that either exposure to chitin or overproduction of the transcription factor TfoX permitted that organism to become competent to take up DNA, leading the way for similar studies in *V. fischeri* (90, 91). Once this capability of *V. fischeri* was established, the types of questions researchers could ask and the rate at which they could be answered were greatly magnified. For example, following the establishment of this new methodology, TfoX-induced competence was cleverly used to identify and map the position and endpoints of a large deletion that arose spontaneously in one lineage of ES114 (92). Mutations marked with an antibiotic resistance cassette could be readily moved from one strain to another by isolating genomic DNA and using it to transform another strain, thus facilitating the ability to “back-cross” and confirm that a phenotype was linked to a mutation of interest and permitting easy generation of strains carrying multiple mutations. Subsequently, a set of tools was generated that facilitated rapid genetic engineering of strains by using PCR SOEing to generate deletion and insertion cassettes that could be introduced into competent cells (93). Specifically, this work developed a set of cassettes with common linker sequences that could be used to generate a family of gene replacement mutants with different antibiotic resistance markers. The markers were flanked by FRT sequences that are recognized by the Flp enzyme that resolves the intervening sequences (94). As a result, the limited number of working resistance cassettes could be used sequentially, then removed to permit their use at another location in the genome. With this technology, the field has generated strains carrying up to seven mutations (95) and a set of 50 individual mutants deleted for genes involved in the metabolism of the second messenger c-di-GMP (96). Similarly, tools that facilitated the insertion of genes at a benign location in the chromosome enabled complementation with wild-type genes as well as derivatives that carried point mutations in sequences of interest, permitting an assessment of genetic requirements for function (93, 97). Together, these advances have developed *V. fischeri* strain ES114 into a robustly tractable microbe that can be easily manipulated to determine underlying mechanisms. While not all *V. fischeri* isolates are as readily engineered, it may be just a matter of time before additional tools are developed, such as one that was recently reported to increase the competence of strain KB2B1 (98).

## THE GENOMICS ERA

**Fig 6:**
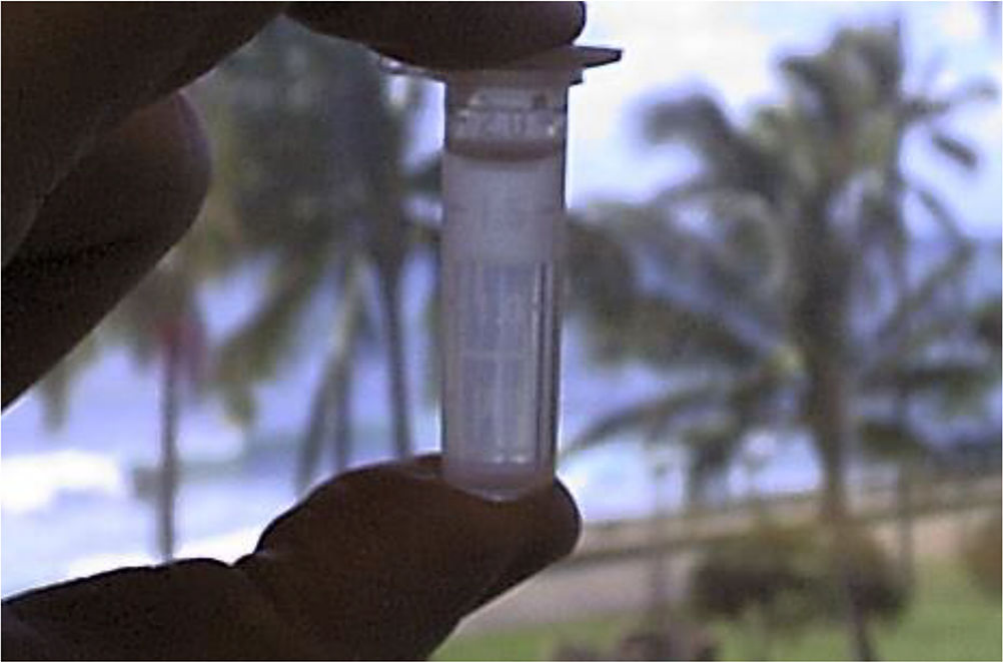
History in the making: the *V. fischeri* genome ready for sequencing. Vial of genomic DNA of *V. fischeri* strain ES114, just prior to submission for sequencing, with Hawaiian landscape in the background. Photo by Eric Stabb, reproduced with permission.

When whole-genome sequencing became feasible in the early 2000s, the field was tasked with identifying a representative *V. fischeri* strain for this analysis. The substantial amount of work probing luminescence control in MJ-1 provided an argument for selecting that strain. However, MJ-1 was isolated and studied during a time when bacteria were maintained by continuous passage on agar plates, rather than being stored in −80°C freezers. During this process for MJ-1, researchers would choose the brightest colonies for subsequent study/restreaking, resulting in the accumulation of multiple versions of MJ-1 of different lineages. Concerns over this in-lab evolution of MJ-1 prompted the selection instead of ES114, a more recent isolate from squid that had not been passaged in the same manner (Fig. 6). By the time it was chosen for sequencing, ES114 had become the “go-to” strain for studies of the *V. fischeri-*bobtail squid symbiosis. This 114th isolate from *E. scolopes* was initially selected for study because it contained a large plasmid that researchers hypothesized could encode symbiosis-specific functions. While that turned out to not be the case (it encoded proteins involved in conjugation), ES114 also had the advantage that it lacked the small plasmids present in other isolates that, if present, could be lost inadvertently or interfere with subsequent introduction of engineered plasmids (99). Ultimately, the decision to sequence a recent isolate would allow high confidence in the utility of genomic information to inform future studies with ES114.

The availability and advancement of genome sequencing technology further expanded the utility of *V. fischeri* as a model organism to better understand how bacteria evolve to compete for and colonize new host niches. Whole-genome sequencing of strain ES114 in 2005 revealed key similarities to the best-studied *Vibrio* species at the time, the pathogen *V. cholerae* (100, 101). Both organisms and other *Vibrio* species, such as the human pathogens *Vibrio parahaemolyticus* and *Vibrio vulnificus*, maintain two chromosomes: a larger one enriched for housekeeping functions and a smaller one enriched for genes thought to promote niche specificity (100, 102). ES114 also has a single large plasmid with genes important for a type IV secretion system that appears to function in conjugation (86). The ES114 genome includes remnants of the cholera toxin phage-like locus, containing a number of those genes but notably missing the *ctxAB* genes that encode the toxin (101, 103). ES114 also includes most, but not all, of the genes for the toxin co-regulated pilus locus, which is a major virulence factor in *V. cholerae* (104).

As more host-associated *Vibrio* genomes were sequenced and integrated into host colonization studies, the resulting similarities between a beneficial symbiont and its relatives from mammalian hosts revealed commonalities in host colonization factors than had previously been noted (105). Indeed, although *V. fischeri* is thought to have been associated with its marine animal host hundreds of millions of years before its congeners began infecting humans (106), many gene-regulation and colonization strategies remain, despite the divergence in host preference. Sequencing of additional *V. fischeri* isolates, from either light organs of diverse hosts or experimental evolution models, revealed how strain-level differences and horizontal gene transfer impact interbacterial interactions, gene regulation, and host specificity [e.g., references (107–112)]. For example, *V. fischeri* strain MJ11, a more recent brightly luminous isolate from the fish *M. japonica*, and other MJ isolates (likely including MJ-1) are deficient for squid colonization (108, 113). Thus, the genomes of MJ11 and additional *V. fischeri* strains paved the way for comparative genomics analyses that have provided insight into how intraspecific genome evolution impacts competition for a specific host niche and host range expansion. We note that the ability to sequence and compare strains also led to the discovery that strain ESR1 (61), a rifampicin-resistant derivative of ES114 that was used for many years to facilitate the recovery of *V. fischeri* following conjugation with *E. coli*, was inadvertently derived from a mutant lineage of ES114 that carried a 10 kb deletion that included genes for glycerol utilization. However, while ESR1 exhibits a competitive colonization defect, its parent with the same deletion does not, indicating that glycerol utilization is not important for early colonization and that other identified changes in the ESR1 genome must account for its colonization defect (92).

## SELECT *V. FISCHERI* TRAITS OF INTEREST BEYOND LUMINESCENCE

### Motility and chemotaxis

Flagella-driven motility and chemotaxis have been intensively studied in bacteria in general and also for their role(s) in pathogenesis. In many cases, motility is an important trait required for bacterial colonization of animal tissue, but often the colonization deficiency of a non-motile mutant is not severe [e.g., references (114–116). *V. fischeri* is remarkable in that motility-defective strains of *V. fischeri* fail to colonize *E. scolopes* (61, 117). To date, flagellar motility remains the only factor that is absolutely required for colonization; other mutants may exhibit severe colonization defects or fail to be retained in the symbiosis, but generally have some level of competence in colonizing, if only transiently. Furthermore, the flagella required for entry are lost following host colonization (118), an observation that implies that they are disadvantageous inside the light organ, perhaps by interfering with growth to a high cell density. Recent work suggests that the flagella are lost via ejection as occurs in *Caulobacter crescentus* (119, 120).

A recent review nicely describes notable motility characteristics of *V. fischeri* (121). *V. fischeri* is somewhat unusual in its production of a tuft of two to seven polar flagella, an arrangement known as lophotrichous (Fig. 5A); most other bacteria have peritrichous (around the cell) or unipolar flagella (118, 122, 123). Another uncommon feature is the presence in the genome of six flagellin genes and the detection in flagella of at least five of these distinct flagellin proteins (124), although this aspect of incorporating more than one flagellin protein is a trait *V. fischeri* shares with other *Vibrio* species [e.g., reference (125)].

Like other marine vibrios, *V. fischeri* is believed to use a sodium-driven flagellar motor (126). Such motors, at marine sodium levels, can produce a high torque (127). This feature may permit *V. fischeri* to more readily migrate through the highly viscous mucus found within the light organ environment (128, 129). *V. fischeri*’s high torque capability has been attributed to its wider flagellar rotor, generated by the presence of two additional stators and supported by one or both of two extra rings (T-ring and H-ring) that surround the flagellar basal body (130).

Another feature of note is the presence of a membrane sheath surrounding the flagella (61). This sheath is believed to derive from the outer membrane, although evidence from other organisms suggests that the sheath diverges in composition relative to the outer membrane (131). In *V. fischeri*, rotation of the sheathed flagella causes the release of LPS, a molecule known to induce host development (68, 132). It also promotes the release of outer membrane vesicles (OMVs) (124, 133). These OMVs contribute to the induction of host development (133). Of note, it was recently discovered that these OMVs can also deliver the bacterial small regulatory RNA SsrA into host epithelial cells, impacting their swelling and calming their immune response (134).

Flagella-driven chemotaxis is also a key aspect of host colonization (60, 135). *V. fischeri* encodes 43 proteins predicted to function as chemoreceptors, a large number compared to the five in enteric bacteria such as *E. coli* (101, 107, 136). Only a few of these have been characterized (38, 137, 138), and none have been demonstrated to recognize chitobiose, a squid-produced chemoattractant that directs *V. fischeri* into the light organ (60, 67). Therefore, many truths remain to be uncovered regarding how *V. fischeri* navigates its environment, both outside and within its host.

### Biofilm and dispersal

The advent of GFP as an optical tool permitted real-time imaging of the interactions of *V. fischeri* with its host, prompting the discovery that this microbe interacted with the surface of its host’s symbiotic organ in the context of a group, or aggregate, of bacteria (59). While other Gram-negative microbes appeared similarly able to form these aggregates, *V. fischeri* readily dominated within aggregates formed from mixed-species inocula, suggesting that this event could be one level at which specificity in the symbiosis is achieved (59). *V. fischeri* remains in the aggregates for short periods of time (up to 3 hours) before exiting or dispersing from them to enter and colonize the light organ (59, 139).

A connection between symbiotic aggregation and biofilm was made when biofilm-relevant genes required for squid colonization were identified and characterized (140, 141). Disruption and overexpression of the regulatory gene *rscS*, which encodes a sensor kinase, prevented and enhanced, respectively, symbiotic aggregate formation and colonization (140, 142). In culture, *rscS* overexpression induced the formation of biofilm phenotypes, including wrinkled colonies (Fig. 5B) and pellicles. These biofilm phenotypes depended on *syp*, a large 18-gene locus that is required for symbiosis and encodes proteins predicted to regulate, produce, modify, and export a polysaccharide, SYP (141, 143). The observed correlations between *in vitro* and *in vivo* biofilm formation as well as host colonization permitted the use of this model system for understanding mechanisms underlying host-relevant biofilm formation.

The *syp* locus is present in other marine bacteria, and particularly conserved in the Vibrionaceae, including in two prominent human pathogens, *Vibrio vulnificus* (*rbd*) and *V. parahaemolyticus* (*scv*), which can be transmitted via contaminated seafood such as raw oysters (141, 144, 145). In contrast, *syp* is absent in the vibrio human pathogen best studied with respect to biofilm formation, *V. cholerae*, which instead relies on a distinct polysaccharide (141, 146). While a role for the *V. vulnificus* locus in mouse virulence was tested, little to no impact was observed (147). This result, combined with the importance of *syp* in squid colonization and the relative conservation of this locus in marine bacteria, may indicate that SYP contributes primarily to microbe-host interactions in the marine environment, such as, potentially, microbe-oyster interactions.

Transcriptional regulation of the *syp* locus and its downstream products have been intensively studied, and a complex regulatory scheme has been uncovered that includes multiple sensor and response regulatory proteins and environmental- and/or squid-relevant signals [for a recent review, see reference (148)]. For example, calcium, which is present in seawater, and nitric oxide, which is found in the context of the light organ, positively and negatively regulate biofilm formation, respectively (97, 149, 150). Given the growing body of literature about the roles of calcium ions and nitric oxide in controlling biofilm formation and dispersal in a variety of bacteria, these findings indicate commonalities in the mechanisms used by pathogens and symbionts alike.

The production of SYP is also regulated at an unknown level by SypA, a single-domain STAS protein. STAS domains are present in diverse bacteria but are best understood in systems where they function either as (i) anti-sigma antagonists [e.g., references (151, 152)] or (ii) components of multiprotein stressosomes (153). However, SypA is unlikely to function in either role (53, 154) and thus joins a growing number of examples in which single-domain STAS proteins exhibit alternative and, in many cases, as yet unknown activities (155).

*V. fischeri* also contains the bacterial cellulose synthesis locus, *bcs*, and uses cellulose to form biofilms on *in vitro* surfaces such as glass and plastic (148). In *E. coli* and *Salmonella*, where cellulose production has been most heavily studied, the second messenger c-di-GMP promotes biofilm formation by binding to and activating cellulose synthase (156, 157). *V. fischeri* likely exerts a similar control over synthase activity but also clearly wields distinct, environmentally responsive regulatory control over *bcs* transcription (158).

Finally, while a substantial body of knowledge now exists with respect to host-relevant biofilm formation, a large gap remains in understanding how *V. fischeri* disperses from its biofilms, either in culture or in the context of aggregates formed on the surface of its squid host. Given that biofilms are problematic in a variety of contexts, including in human infectious disease, due, in part, to their tolerance to antimicrobials, it is important to understand how host-relevant dispersal is triggered. Some insight was garnered recently by the identification of *V. fischeri* genes that direct synthesis, secretion, and, ultimately, cleavage from the surface of a large adhesive protein, LapV (159). Disruption of genes that would prevent LapV cleavage caused biofilms to form, suggesting that those laboratory conditions normally promote continual dispersal (159). This work thus provided the first insight into dispersal by *V. fischeri* and a foundation for additional studies.

### Competition

Although it is well known that competitive exclusion prevents multiple species from occupying the same niche, this ecological theory has been challenging to study within animal hosts. Recent work in other model systems revealed that competition for space and resources can be most fierce within a species (160), suggesting that intraspecific competition may have important ecological implications for structuring a host-associated microbiome. Indeed, work by Bongrand et al. (110) showed that closely related *V. fischeri* symbiont isolates evolved a competitive mechanism to occupy the squid host niche and suppress subsequent colonization by other symbiotic populations. This mechanism, which involves rapid colonization of the light-organ crypts, has been described in a recent review (161). This discovery of intraspecific competitive dominance behavior among *V. fischeri* populations expanded the utility of this organism as a model in which to study competition in the context of a beneficial symbiosis.

Although the competitive dominance phenotype observed in some *V. fischeri* strains provides a colonization advantage, this behavior is not exhibited by a majority of isolates examined, which tend to colonize the light organ crypts at the same time. Such co-colonization events might act as a strong selective pressure for *V. fischeri* symbionts to evolve one or more interference competition mechanisms to directly kill or stop the growth of a competitor in the same crypt. Studies using strains FQ-A001 and ES401 demonstrated that a strain-specific, type VI secretion system (T6SS2) interbacterial weapon (Fig. 5C) is necessary to prevent co-colonized crypts and outcompete a T6SS-deficient competitor in the light organ (112, 162, 163). By using fluorescence microscopy to localize each competitor within the crypt spaces, these data also revealed that T6SS-mediated killing is also capable of spatially structuring a host-associated microbiome through interference competition. Originally described in the pathogen *V. cholerae* (164), studies exploring the T6SS in *V. fischeri* add to our knowledge of how this weapon is used in the context of a beneficial bacterial-animal association (165, 166).

Using *V. fischeri* as a model to study T6SS-mediated competition also fostered a breakthrough in understanding how cells mediate the cell-cell contact required for the transfer of cytotoxic effector molecules. Although T6SS gene expression and aggregation are not apparent in *V. fischeri* cells grown in standard liquid media (LBS), increasing the viscosity using a polymer to produce a hydrogel that mimics the host environment activated T6SS expression, assembly, and function and resulted in the aggregation of competing strains via a heterotypic interaction that requires a putative lipoprotein (163). This aggregation was specific (only certain species were integrated and killed with T6SS) and necessary for competition in the host (163). These studies help shape our understanding of how and where the T6SS mechanism may impact host-associated microbiomes, both in beneficial and pathogenic interactions.

It is also notable that, of the >70 *V*. *fischeri* isolates and genomes characterized, none appear to carry a diffusible killing mechanism, despite it being common among marine vibrios (167). The curious lack of a diffusible weapon may indicate such a competitive strategy is not tolerated in the context of the symbiosis. Indeed, *V. fischeri*-produced diffusible molecules, such as autoinducer, are capable of disseminating between crypts, and studies have shown that antibiotics can diffuse into the light organ to kill resident symbiont cells that lack engineered resistance [e.g., reference (168)]. What might be the evolutionary pressure(s) to select against diffusible weapons, yet select for contact-dependent strategies? *V. fischeri* exists in other marine niches, including in the gut tracts of fishes, estuaries, and within the plankton (2, 45, 169, 170), where selection also likely occurs and may drive the need for evolutionary trade-offs. Future work may reveal how the combination of ecological interactions and evolutionary pressures, or eco-evo dynamics, have influenced the evolution of *V. fischeri* genomes and the strategies they use to compete for symbiotic and other niches.

## SERENDIPITOUS EVENTS

Like other bacterial model systems, there have been serendipitous events in the history of *V. fischeri* that have greatly impacted the utility of the system. For example, the choice of ES114 as an initial focal strain certainly set a course for discoveries that may have been quite different if another strain had been chosen. With >70 *V*. *fischeri* genomes now sequenced and more labs exploring inter-strain variation, strain-specific differences have been reported for a variety of phenotypes, including T6SS content and effectors, regulation of biofilm and luminescence, aggregation size, and speed of host colonization [e.g., references (27, 112, 139, 171–173)]. Strain ES114 was selected initially because of its plasmid characteristics, but it was the genetic tractability of this strain that allowed for the rapid development of genetic tools that facilitated answering questions about bacterial physiology, interbacterial interactions, and host colonization. It was not until later, when researchers began exploring the diversity within *V. fischeri*, that they could appreciate just how tractable ES114 is. Although the genetic tools developed using ES114 have been applied to multiple *V. fischeri* isolates, and even used to manipulate other species [e.g., reference (174)], it is common knowledge among *V. fischeri* research groups that some strains are more challenging to genetically manipulate. It was, therefore, a fortunate accident that ES114 was selected as a focal strain early in the development of this model.

Another serendipitous event was the selection of the standard growth medium for *V. fischeri*. Luria-Bertani (LB) broth is a commonly used complex medium for growing enteric bacteria, and researchers found that by simply increasing the sodium chloride to accommodate a marine bacterium, a buffered version of this medium termed LBS worked well to cultivate *V. fischeri* strains (175, 176). What has become apparent over the years is that LBS liquid broth is an excellent “off” condition for many ecologically relevant functions in *V. fischeri*. This medium has sufficient iron to repress siderophore and iron uptake pathways (177) and does not permit cells to fully induce luminescence (178), biofilm formation (56), T6SS and aggregation (55, 179), or natural transformation (91). This “off” condition allowed researchers to modify the media to find ecologically relevant cues and signals that activate or repress functions and study natural regulatory pathways important for host colonization and survival. Appreciating that this medium may be an “off” condition for other traits could facilitate a deeper understanding of additional processes in the future.

## FUTURE DIRECTIONS

Past work has built a strong foundation of knowledge and tools in the areas of imaging, genetics, genomics, host colonization, motility, biofilm, interbacterial communication and competition, and others. Future work will find connections between these subfields and build out a broader ecological and evolutionary understanding of how individual *V. fischeri* cells sense their environment and coordinate behavior to enhance their survival. It will also take advantage of applying new methods to answer questions that were not previously possible to address. Below we outline some of the exciting frontiers to which we expect *V. fischeri* to contribute.

How do cells integrate multiple and sometimes conflicting regulatory cues/signals to control functions? Just as the cellular redox state can repress quorum sensing despite cells being at high cell density (30), so too will hierarchies of cues be identified and resolved for additional behaviors. Indeed, many different activating and repressing cues/signals have been identified that regulate biofilm, quorum sensing, T6SS, and motility, and we expect *V. fischeri* to have evolved complex logic gates to regulate and balance these important behaviors to optimize success. Discovering these logic gates will reveal specific conditions where regulated functions are beneficial to the cell and could provide novel tools for gene regulation in synthetic biology.

What are the metabolic costs of critical host-associated functions and how do these costs impact eco-evo dynamics? For example, bioluminescence is an energy-requiring chemical reaction, and although dim or dark strains grow faster than their bright counterparts in culture, dark mutants cannot persist in the host light organ (73, 74, 180). These findings suggest that there is selective pressure in the natural host to maintain an energetically costly function. Future studies of the energetic and metabolic costs of this and other host-relevant behaviors can be further explored in *V. fischeri*. Once we have established the regulatory cues and metabolic costs of these functions, the selective pressures driving their maintenance or loss while in the host can be further explored.

How do different strains solve the same problem? Work to date has already revealed a diversity of strains with different abilities to form biofilms and compete for colonization. Genes that are critical in one strain, such as RscS, may be absent or altered in other strains, leading to a re-routing of regulatory pathways that may be informative to a more global view of host colonization (108, 172). Adaptation of additional tools and techniques, including CRISPRi [now in progress (181)] and RNAi, could facilitate the analysis of strains of interest that are less genetically tractable to explore the diversity of mechanisms that can drive the underlying phenotype.

Recent innovations in imaging technology will allow researchers to fill in knowledge gaps at a finer scale than has previously been possible to understand micro-scale biogeography and its consequences to the bacteria. Previous work has shown that the spatial context of symbiotic *V. fischeri* matters. For example, two different genotypes can be present in the same light organ but be either well-mixed or spatially separated in the host (139, 182), and *V. fischeri* cells in certain microhabitats express different genes (63–65). Recent advances in cryoEM (183, 184) and spatial metabolomics (185) can provide unprecedented details of symbiotic *V. fischeri* cells interacting with either one another or host cells, along with the chemical landscape.

Studies of *V. fischeri* can also contribute to the question of how bacteria can be leveraged to solve medical problems. The *V. fischeri*-squid model continues to deliver insights on microbe-host interactions. The finding that *V. fischeri*-produced OMVs can deliver small regulatory RNAs into host cells (134) provides a foundation for further mechanistic studies that have the potential to foster practical applications such as medical treatments of specific diseases. The study of *V. fischeri* may also help address the looming crisis of widespread antibiotic resistance by developing a deeper understanding of resistance in the context of a natural animal host. Recent work revealed that host-associated *V. fischeri* cells are less susceptible to the antibiotic chloramphenicol than those grown in culture and that re-bound growth of antibiotic-exposed symbionts stemmed from “resilient” bacteria present in crypt 3, the smallest, least developed crypt (168). Understanding host-associated dynamics like these may yield new strategies for the treatment and control of reservoir bacterial populations.

Finally, the use of generative artificial intelligence (AI) and machine learning is expected to accelerate knowledge discovery in most research fields, including microbial symbiosis. With researchers quickly amassing data using genomic, transcriptomic, proteomic, imaging, and high-throughput experiments, these data can be analyzed and explored with machine and deep learning approaches to make new connections and generate testable hypotheses at a pace that was not previously possible. Moreover, recent progress in predicting protein structures and functions based on DNA sequences alone has offered a glimpse into how the trove of genes in microbial genome sequences might one day be screened for functions of interest. Indeed, a recent paper that used a deep learning approach to screen 12 million commercially available molecules for treatment against methicillin-resistant *Staphylococcus aureus* identified over 200 candidates to test in the lab, ultimately yielding two new drugs that were effective at killing this notorious pathogen (186). Future applications of AI and machine learning will no doubt reveal new insight into how *V. fischeri*, and other model organisms, evolve and interact with ambient molecules, as well as other microbial and eukaryotic cells.

## CONCLUDING REMARKS

*V. fischeri* itself initially offered two key drivers of interest: its abilities to produce light and form an exclusive beneficial symbiosis in an animal host. These drivers were amplified when paired with (i) a host that is accessible, amenable to imaging, and with microbe-responsive behaviors and biogeography that informs microbial sensory transduction, and (ii) innovative researchers who pushed the envelope in conceptual and technological advances to study microbe-host interactions and who attracted collaborators in diverse disciplines to apply novel approaches that yielded new perspective and understanding. The result is a model microbe that continually yields insights into a variety of microbial processes well beyond the initial features of interest and relevant across the spectrum of behaviors in bacteria-host interactions.
